# Within-Host Phenotypic Evolution and the Population-Level Control of Chronic Viral Infections by Treatment and Prophylaxis

**DOI:** 10.3390/math8091500

**Published:** 2020-09-04

**Authors:** Dmitry Gromov, Ethan O. Romero-Severson

**Affiliations:** 1 Faculty of Applied Mathematics and Control Processes, Saint Petersburg State University, St. Petersburg 199034, Russia; 2 Department of Mathematics, National Research University Higher School of Economics, St. Petersburg Campus 16 Soyuza Pechatnikov Str., St Petersburg 190121, Russia; 3 Theoretical Biology and Biophysics Group, Los Alamos National Laboratory, Los Alamos, NM 87545, USA

**Keywords:** multi-strain infectious diseases, mathematical modeling, basic reproduction number, sensitivity analysis

## Abstract

Chronic viral infections can persist for decades spanning thousands of viral generations, leading to a highly diverse population of viruses with its own complex evolutionary history. We propose an expandable mathematical framework for understanding how the emergence of genetic and phenotypic diversity affects the population-level control of those infections by both non-curative treatment and chemo-prophylactic measures. Our frameworks allows both neutral and phenotypic evolution, and we consider the specific evolution of contagiousness, resistance to therapy, and efficacy of prophylaxis. We compute both the controlled and uncontrolled, population-level basic reproduction number accounting for the within-host evolutionary process where new phenotypes emerge and are lost in infected persons, which we also extend to include both treatment and prophylactic control efforts. We used these results to discuss the conditions under which the relative efficacy of prophylactic versus therapeutic methods of control are superior. Finally, we give expressions for the endemic equilibrium of these models for certain constrained versions of the within-host evolutionary model providing a potential method for estimating within-host evolutionary parameters from population-level genetic sequence data.

## 1. Introduction

Pathogens that lead to persistent chronic infection in people must mitigate both the innate and adaptive immune systems. Strategies for evading the innate immune system are complex including direct subversion of host signaling pathways [1]. Pathogens such as HIV avoid the adaptive immune system by simply evolving new phenotypes faster than the host immune system can adapt, leading to a rapid co-evolutionary race. Because HIV has a short generation time and generates a massive number of new viral particles each day [2], this evolutionary race creates a large potential for the emergence of new viral strains and phenotypes. This rapid evolutionary process is one of the many reasons that HIV is exceedingly difficult to treat. Mutations that evolve in a single host are also known to be transmitted. In 2014–2016, 6 out of 11 countries looking for the presence of pre-treatment drug resistance (i.e., presence of a drug resistant phenotype in persons unexposed to the drug) reported greater than 10% of new infections were resistant to one or more non-nucleoside reverse-transcriptase inhibitor, which is related to both treatment failure and death [3]. The rapid emergence of new viral phenotypes within infected persons is not just a clinical problem, it is an epidemiological problem. Chemo-prophylactic measures that focus on protecting uninfected persons using similar drugs to those used for treating infected persons are not immune to evolutionary derailment. In King Country, Washington, 0.5% of people living with HIV were found to have resistance mutations to the drugs used for prophylaxis [3]. However, with the emergence of chemo- and bio-prophylactic agents (i.e., anti-HIV antibodies for prevention), we must consider the possibility that population-level administration of these agents can shift the ever evolving landscape of chronic viral infections to more resistant variants.

This paper is motivated by a need for mathematical models that integrate within-host genetic diversity and phenotypic evolution with epidemiological dynamics and consider the effects of joint therapeutic and prophylactic controls. We also attempted to balance the complexity of the model to be usable as a data analysis tool with the desire to understand the mathematical and statistical properties of the model using analytical methods. Our model accounts for within-host evolution among multiple phenotypes characterized by variable contagiousness, resistance to prophylactic measures, and resistance to therapeutic measures. Our framework allows for new phenotypes to emerge in chronic infection that can be both transmitted and possibly lost in later hosts. We consider both the epidemiological and evolutionary effects of both therapy for infected persons and chemo-prophylaxis-type measures for uninfected persons.

There has been a number of results devoted to the analysis of different aspects of the evolutionary and epidemiological dynamics of a multi-strain pathogen. While there is a wide spectrum of different models covering different aspects of virus/immune system evolution and their interaction, most developed models are too complex to be analyzed analytically thus, their analysis restricts to carrying out and analyzing the results of numerical simulations. Our model is related to the approach of Lythgoe et al. [4] that considers the possibility of a person infected with a virus of type *i* can transmit a virus of type *j* at a time-dependent rate *β_ij_*(*t*). While this approach presents a detailed model of the within-host viral evolution, it requires a substantial amount of data that is not readily available: virus reproduction, mutation and death rates. Furthermore, since we need to take into account the duration of the infection at the time when transmission occurs, the system dynamics is governed by integro-differential equations, which are difficult to deal with. On the other hand, such a detailed approach turns out to be an overkill as the total pool of infected contain the individuals at different stages of disease and hence, the transmission rates undergo a sort of averaging over the whole set of infected. Therefore, we employ a simpler formalism in which we treat the virus evolutionary dynamics in a more coarse grained fashion. This allows us to balance our mutual goals of a sufficiently complex model that can still be approached analytically.

Complex multi-strain models have been proposed for influenza [5,6] and dengue [7] that focus on both cross-reactivity among circulating strains and coinfection [8] rather than the emergence of new strains within infected hosts. Much of this work is often based on complex models that are intended to explain specific biological phenomena that are too complex to be understood by applied analysis methods. On the other hand, there has been a number of papers devoted to the analytic analysis of certain aspects of multi-strain virus dynamics. However, most of the papers either deal with rather restricted setups or study only certain aspects of the system dynamics. We mention stability analysis of within-host multi-strain virus dynamics with mutations [9]; analysis of a multi-strain (actually two-strain) disease with environmental transmission, no mutations [10]; bifurcation analysis of a number of (rather simple) multi-strain epidemiological models without mutation [11].

Further information about different approaches to modeling the evolutionary and population-based dynamics of multi-strain pathogens as well as the description of the problems that arise in this connection can be found in [12,13]

It should be noted that most research effort aimed at studying the dynamics of multi-strain viruses does not take into account the possibility of within-host mutations and concentrate on modeling different immune system responses in reaction to re-infection or coinfection. In contrast to that, we are more concerned with the effect of mutations on both the within-host and population-level distribution of viral strains and on how both the emergence and loss of phenotypes within infected persons alters the population-level control of chronic viral infections.

## 2. Description of the Models and Their Structural Properties

### 2.1. A Baseline Model of a Chronic Multi-Strain Virus Infection

In the baseline version of the model we consider the within-host evolution and transmission of distinct strains that have the same phenotype. This type of neutral genotypic evolution is the basis of both the molecular epidemiology and phylodynamics of viral infections. In order to account for virus variability, the whole set of viruses is divided into *n* types (strains), *V_i_*, *i* = 1, … , *n* (in the following, we will use the words virus strain and virus type interchangeably). This space defines the evolutionary domain of the model (i.e., only these pre-specified strains can exists); however, the analytical results we present are valid for any number of strains. We assume that all infections are founded by a single virus of type *i*. During the acute infection stage, we assume that the patient’s viral population consists of only the founding-type virus, while during the chronic stage, the original virus is allowed to mutate thus producing new strains according to the within-host evolutionary model. Therefore, we assume that each chronically infected individual’s viral population contains a distribution of viral strains dependent on both the founding viral type and the time from infection.

To model the population-level process of disease propagation we assume an SI model with two stages of disease: an initial acute stage followed by a longer chronic stage. Furthermore, we extend the set of state variables to include the individuals enrolled into treatment. In doing so we assume that the treatment is completely efficient and the patients are fully compliant with the treatment.

When writing the differential equations of the model we assume the inflow to be equal to the outflow hence, the total population size remains constant. Therefore, we write the model equations for the fractions of the respective cohorts in the total population. This implies, in particular, that the sum of all the states is equal to 1. We have the following set of DEs (state variables and parameters defined in Table 1):

(1) $${\dot{I}}_{Ai}=\phi_{i}(\;X)S-\gamma I_{Ai}-\mu I_{Ai}\;{\dot{I}}_{Ci}=\gamma I_{Ai}-u_{T}I_{Ci}-\mu I_{Ci}\;\;\dot{T}=u_{T}\sum_{i=1}^{n}I_{Ci}-\mu T\;\;\dot{S}=\mu -\sum_{i=1}^{n}\phi_{i}(\;X)S-\mu S$$

where X = [*I*_*A*1_, … , *I*_*An*_, *I*_*C*1_, … , *I*_*Cn*_*, T, S*] is the (2*n* + 2)-dimensional state vector, *i* ranges from 1 to *n*, and the respective forces of infection are defined as

(2) $$\phi_{i}(\;X)=\beta_{A}I_{Ai}+\beta_{C}\sum_{j=1}^{n}\alpha_{ij}I_{Cj}.$$

In Equation (2), *α_ij_* ∈ [0, 1] denotes the average fraction of type *i* viruses in the viral population of an individual initially infected by the type *j* virus. It should thus hold that $\sum_{i=1}^{n}\alpha_{ij}=1$ for all *j* = 1, … , *n*. Furthermore, we assume that *α_ii_* ≠ 0 for all *i* = 1, … , *n*. This means that the viral population of an individual infected with the type *i* virus always contains a non-zero amount of the corresponding strain. Parameters *β*_*A*_ and *β_C_* are the transmission rates of acute and chronically infected individuals. In the baseline model, the viruses are phenotypically homogeneous, therefore the probability that a susceptible individual contracts a disease depends only on the disease stage of the infected contact, but not on the type of virus. That is to say, a susceptible can be equally well infected by any virus.

Furthermore, we assume a homogeneous contact structure, i.e., we assume that a susceptible individual can have contact with any infected person with the same probability. The only distinction is made between acutely and chronically infected individuals that are assumed to have different transmissibility coefficients *β*_*A*_ and *β*_*C*_. Specifically, we assume that *β*_*A*_ and *β*_*C*_ differ by a proportionality coefficient *ξ*: *β*_*A*_ = *ξβ*_*C*_ (we shall still occasionally write *β*_*A*_ if it makes the notation more straightforward). With this, expression Equation (2) turns into

(3) $$\phi_{i}(\;X)=\beta_{C}\left(\xi I_{Ai}+\sum_{j=1}^{n}\alpha_{ij}I_{Cj}\right).$$

### 2.2. A Generalized Model with Differentially Effective Control, Variable Transmissibility and Prophylaxis

We generalize the baseline model by allowing different strains to have different phenotypes by relaxing the model assumptions along the following lines:

- The efficacy of the treatment program depends on the viral strain. That is, the treatment program fails with certain probability, which varies depending on the virus strain, causing the treated individuals to thus revert back to active chronic infection.
- Virus strains have different levels of contagiousness.
- The efficacy of prophylactic measures depends on the viral strain. While on prophylaxis, an individual acquires protection against the virus depending on the specific viral strain.

To account for different failure rates of treatment we divide the group of people on treatment into *n* compartments *T*_*i*_, where *i* corresponds to the virus strain. Furthermore, we add a cohort of people receiving prophylaxis, denoted by *P*. While on prophylaxis, the individuals acquire variable protection against different virus strains denoted by *ψ_i_* ∈ [0, 1] with *ψ_i_* = 1 corresponding to full protection. Thus we have the following model:

(4) $${\dot{I}}_{Ai}=\phi_{i}(\;X)S+(1-\psi_{i})\phi_{i}(\;X)P-\gamma I_{Ai}-\mu I_{Ai}\;{\dot{I}}_{Ci}=\gamma I_{Ai}+\zeta_{i}T_{i}-u_{T}I_{Ci}-\mu I_{Ci}\;\;{\dot{T}}_{i}=u_{T}I_{Ci}-\zeta_{i}T_{i}-\mu T_{i}\;\;\dot{S}=\mu -u_{P}S-\sum_{i=1}^{n}\phi_{i}(\;X)S+\delta P-\mu S\;\;\dot{P}=u_{P}S-\sum_{i=1}^{n}(1-\psi_{i})\phi_{i}(\;X)P-\delta P-\mu P$$

where *ζ*_*i*_ ≥ 0 is the failure rate associated with the *i*th control, *δ* is the inverse duration of prophylaxis, and *u*_*T*_, resp., *u*_*P*_ are the rates at which people are administered to either treatment or prophylaxis. To account for variable transmissibility of different virus strains we define a set of transmissibility rates *β*_*Ai*_ and *β_Ci_*, *i* = 1, … , *n*. Similarly to the baseline case, the transmissibility rates for the corresponding acute and chronic stages are assumed to be proportional, i.e., *β*_*Ai*_ = *ξβ*_*Ci*_. The proportionality coefficient *ξ* does not depend on the virus type *i* and is determined by the number of virions in the blood, which is assumed to be the same for all virus types. The forces of infection *ϕ_i_*(*X*) are defined as

(5) $$\phi_{i}(X)=\beta_{Ci}\left(\xi I_{Ai}+\sum_{j=1}^{n}\alpha_{ij}I_{Cj}\right).$$

Note that setting either *ζ_i_* = 0 or *ψ*_*i*_ = 0 or *β*_*Ci*_ = *β*_*C*_ for all *i* = 1, … , *n*, we obtain different variations of the baseline model.

**Notation.** We let **0**, **1**, and E denote the matrices of zeros, ones, and the identity matrix (the use of notation E instead of I for the identity matrix is common in German and Russian mathematical texts (Germ., Einheitsmatrix); here we use it to avoid confusing notation I with the letter *I* used for infected compartments). The sizes of the respective matrices are indicated as subscripts. A single subscript, for example, as in E_*n*_, denotes a square [*n* × *n*] matrix of respective type. Furthermore, I_*A*_ and I_*C*_ denote the column vectors of respective variables and A denotes the matrix of *α*’s:

(6) $$I_{A}=\left[\begin{array}{c} I_{A1}\\ \vdots \\ I_{An} \end{array}\right],\;\;I_{C}=\left[\begin{array}{c} I_{C1}\\ \vdots \\ I_{Cn} \end{array}\right],\;\text{and A}=\left[\begin{array}{ccc} \alpha_{11} & \cdots & \alpha_{1n}\\ \vdots & \ddots & \vdots \\ \alpha_{n1} & \cdots & \alpha_{nn} \end{array}\right].$$

Note that A is a non-negative, column stochastic matrix, i.e., all its columns sum to 1. Necessary facts about special classes of matrices that will be used throughout the text are presented in Appendix A.

All parameters and variables used in the model are listed in Table 1. Note that all quantities used are assumed to take on non-negative values and the index *i* always runs from 1 to *n*.

### 2.3. Structural Analysis

In this subsection we consider only the baseline model Equation (1) since the extended model has the same properties and can be readily analyzed along the same lines.

**Non-negativity of the solutions.** The Equation (1) can be written as

(7) $$\frac{d}{dt}\left[\begin{array}{c} I_{A}\\ I_{C}\\ T\\ S \end{array}\right]=\left[\begin{array}{c} \beta_{C}\;[\xi \;I_{A}+A\;I_{C}]\;S-(\gamma +\mu )\;I_{A}\\ \gamma \;I_{A}-\mu \;I_{C}\\ -\mu T\\ \mu -\beta_{C}1_{\left[1\times n\right]}\;[\xi \;I_{A}+A\;I_{C}]\;S-\mu S \end{array}\right]+\left[\begin{array}{c} 0_{\left[n\times 1\right]}\\ -\;I_{C}\\ 1_{\left[1\times n\right]}\;I_{C}\\ 0 \end{array}\right]u_{T}=\Psi (X)+\Psi^{u}(X)u_{T}.$$

The vector-valued functions Ψ(X) and Ψ^*u*^ (X) are *essentially non-negative*, i.e., for all *j* = 1, … , *m*, *m* = 2*n* + 2, it holds that $\Psi_{j}(\tilde{X})\geq 0$ (resp., $\Psi_{j}^{u}(\tilde{X})\geq 0$) for any $\tilde{X}\in R_{\geq 0}^{m}$ such that ${\tilde{X}}_{j}=0$ (see [14] for details). This implies that solutions of Equation (1) are non-negative. That is to say, for any non-negative initial condition $X(0)=X_{0}\in R_{\geq 0}^{m}$ and any non-negative control *u_T_* the solution of Equation (1) belongs to $R_{\geq 0}^{m}$ for all *t* ≥ 0.

**Boundedness of solutions.** Observe that the *m*-simplex Δ_*m*_, formed as the convex hull of *m* unit vectors **e**_*j*_, *j* = 1, … , *m*, is invariant with respect to Equation (1):

$$X(0)\in \Delta_{m}\Rightarrow X(t)\in \Delta_{m},$$

where $\Delta_{m}=\{\;X\in R_{\geq 0}^{m}|\sum_{j=1}^{m}X_{j}=1\}$. This result follows immediately from the fact that the states *X_i_* represent the fractions of the respective groups within the total population and hence sum to 1.

## 3. Local Analysis at a Disease-Free Equilibrium

Below, we will compute the basic reproduction number for both the baseline and extended models and present a number of related results. To distinguish between the basic reproduction numbers related to different models we will add a superscript denoting the particular model: *α* for the baseline model Equation (1) and *β* for the extended model Equation (4).

### 3.1. Basic Reproduction Number for the Baseline Model

The system Equation (1) has a unique disease-free equilibrium (DFE) X_*DFE*_ = [0, … , 0, 1]. To analyze the stability property of the system Equation (1) at the DFE we compute the controlled basic reproduction number *R*_0_ using the classical next-generation method [15] (see [16] for an extension of the method that takes into account the action of a control).

**Theorem 1.** *For any choice of parameters α_ij_* ≥ 0 *such that* Σ_*i*_ *α_ij_* = 1 *and α_ii_* ≠ 0 *for all i, j* = 1, … , *n, the controlled basic reproduction number of the system* Equation (1) *is given by*

(8) $$R_{0}^{\alpha }(u_{T})=\beta_{C}\frac{\xi (u_{T}+\mu )+\gamma }{\left(\gamma +\mu )(u_{T}+\mu \right)}=\frac{\xi \beta_{C}}{\gamma +\mu }+\frac{\beta_{C}\gamma }{\left(\gamma +\mu )(u_{T}+\mu \right)}.$$

**Proof.** See Appendix B. □

Note that the *α_ij_* values do not affect the basic reproduction number, which makes sense in this context as mutation from one strain into another does not imply any change in a relevant phenotype such as contagiousness or resistance to therapy. In this context, a different strain simply carries a distinct mutation (or pattern of mutations) that makes it identifiable from other strains. However, understanding the distribution of strains with the same phenotype is an important aspect of molecular epidemiology, which is dependent on the specific *α_ij_* values. This relationship between within-host mutations and endemic equilibrium of infection types is discussed below.

**Sensitivity analysis.** When devising an intervention strategy, the main question to be answered is whether the proposed treatment or prophylaxis scheme is capable of eliminating the infection, i.e., driving the basic reproduction number below 1. To address this issue we introduce the sensitivity parameter(s) *R*_1_ that quantify the efficiency of sufficiently small controls in reducing the value of *R*_0_, [16]. In particular, the controlled basic reproduction number $R_{0}^{\alpha }(u_{T})$ is expanded as

(9) $$R_{0}^{\alpha }(u_{T})=R_{0}^{\alpha }+R_{1}^{\alpha }u_{T}+O(u_{T}^{2}),$$

where $R_{0}^{\alpha }=R_{0}^{\alpha }(0)=\beta_{C}\frac{\gamma +\xi \mu }{\mu (\gamma +\mu )}$, $R_{1}^{\alpha }=-\frac{\beta_{C}\gamma }{\mu^{2}(\gamma +\mu )}$, and $O(u_{T}^{2})$ is a high-order term, which is proportional in magnitude to the square of the control *u_T_*. Before proceeding with further analysis, we define the notion of efficiency of a control.

**Definition 1.** *Let the uncontrolled basic reproduction number be larger than 1, i.e., R*_0_ (0) > 1. *A control u is said to be*

1. *Locally efficient if the respective sensitivity parameter is negative, i.e., R*_1_ < 0*;*
2. (Globally) efficient if there exists a non-negative value u* *such that R*_0_ (u*) = 1.

Furthermore, we say that a control is unconditionally locally (globally) efficient if 1. (2.) holds for all admissible values of parameters. Otherwise the control is said to be conditionally efficient.

We can immediately observe that *u_T_* is unconditionally locally efficient. However, an unconditionally locally efficient control may fail to reach the stated goal of eliminating the infection, i.e., reducing *R*_0_ below 1. The following result illustrates that.

**Lemma 1.** *The control u*_*T*_ *is globally efficient if β_C_ satisfies*

(10) $$\xi \beta_{C}<\gamma +\mu .$$

**Proof.** This result can be easily checked by observing the expression for *R*^*α*^ (*u*_*T*_) in Equation (8) and noting that the second summand vanishes as *u_T_* tends to infinity. □

**Remark 1.** *Note that the condition Equation (10) can be alternatively rewritten as β*_*A*_*θ*_*A*_ < 1, *where θ*_*A*_ = 1/(*γ* + *μ*) *denotes the average duration of the acute stage*.

The result of Lemma 1 implies that the control *u_T_* is only *conditionally* globally efficient. That is, it can be used to completely eliminate the infection only if the transmissibility *β_C_* satisfies Equation (10).

### 3.2. Basic Reproduction Number for the Extended Model

In contrast to the baseline case, the disease free equilibrium for the modified model Equation (4) is shifted due to the action of the control *u_P_*. So, we have

(11) $$X_{DFE}=[0_{1\times n},\;0_{1\times n},\;0_{1\times n},\;P_{DFE},\;S_{DFE}],$$

where $S_{DFE}(u_{P})=\frac{\delta +\mu }{\delta +\mu +u_{P}}$ and $P_{DFE}(u_{P})=1-S_{DFE}(u_{P})=\frac{u_{P}}{\delta +\mu +u_{P}}$. Local stability of the DFE Equation (11) is determined by $R_{0}^{\beta }(u_{T},u_{P})$. Before we proceed with the analysis, we note that the results to follow will be formulated using matrix notation. In particular, we will write B_*C*_ = diag(*β*_*C*1_, … , *β_Cn_*), Ψ = diag(*ψ*_1_, … , *ψ_n_*), and *Z* = diag(*ζ*_1_, … , *ζ_n_*) to denote the diagonal matrices of transmissibility rates, protection factors and treatment failure rates.

**Theorem 2.** *The controlled basic reproduction number of the system* Equation (4) *is given by*

(12) $$R_{0}^{\beta }(u_{T},u_{P})=\frac{{\bar{\beta }}_{C}(\gamma +\xi \mu )}{(\gamma +\mu )\mu }\rho \;(Q(u_{P})N(u_{T})),$$

*where* ${\bar{\beta }}_{C}=\max_{i}\;\beta_{Ci}$, ${\bar{B}}_{C}={\bar{\beta }}_{C}^{-1}\;B_{C}$, $Q(u_{P})={\bar{B}}_{C}\;[E_{n}-P_{DFE}(u_{P})\Psi ]$, $N(u_{T})=\frac{1}{\gamma +\xi \mu }[\xi \mu \;E_{n}+\gamma A\Delta (u_{T})]$, *and* Δ(*u*_*T*_) = (Z + (*μ* + *u*_*T*_) E_*n*_)^−1^ (Z + *μ* E_*n*_).

**Proof.** See Appendix B. □

Note that the basic reproduction number of the extended system is a product of two terms: the first one closely resembles $R_{0}^{\alpha }$ as in Equation (9), while the second term is the spectral radius of the product of two matrices, where the first one depends only on *u*_*P*_ and the second one depends only on *u*_*T*_.

Before we proceed with the analysis, we formulate an important result on stochastic matrices that we need to obtain further results.

**Lemma 2.** *Let* Σ *be a non-negative, column stochastic matrix. Then for any α* ∈ [0, 1], *the convex combination* Σ_*α*_ = *α* E + (1 – *α*)Σ *is a column stochastic matrix as well. Furthermore, the left and right dominant eigenvectors of* Σ *coincide with those of* Σ_*α*_.

**Proof.** Consider the *i*th column of the matrix Σ_*α*_, *i* = 1, … , *n*. Summing its components and using the fact that Σ is column stochastic we get *α*1 + (1 – *α*)1 = 1. This implies that Σ_*α*_ is column stochastic as well. Further, let *v* be the right dominant eigenvector of Σ, i.e., Σ*v* = *v*. We have

$$\Sigma_{\alpha }v=\alpha \;Ev+(1-\alpha )\Sigma v=\alpha v+(1-\alpha )v=v,$$

hence, it is the right dominant eigenvector of Σ_*α*_ as well. The case of a left dominant eigenvector is shown analogously. □

**Sensitivity analysis.** We begin this paragraph by writing down an expansion of $R_{0}^{\beta }(u_{T},u_{P})$.

**Theorem 3.** *Let* A *be irreducible and let w*_0_ *and v*_0_ *be the left and the right dominant eigenvectors of* $Q(0)N(0)={\bar{B}}_{C}\;\bar{A}$, *corresponding to* $\rho \;(\;{\bar{B}}_{C}\;\bar{A})$ *and normalized such that* $w_{0}^{T}v_{0}=1$. *The controlled basic reproduction number* $R_{0}^{\beta }(u_{T},u_{P})$ *can be written as*

(13) $$R_{0}^{\beta }(u_{T},u_{P})=R_{0}^{\beta }+R_{1,T}^{\beta }u_{T}+R_{1,P}^{\beta }u_{P}+O(\|(u_{T},u_{P}){\|}^{2}),$$

*where* $R_{0}^{\beta }\;\;\;=\;\;\frac{{\bar{\beta }}_{C}(\gamma +\xi \mu )}{(\gamma +\mu )\mu }\rho (\;{\bar{B}}_{C}\;\bar{A})$, $R_{1,T}^{\beta }\;\;\;=\;\;\;-w_{0}^{T}\left[R_{0}^{\beta }\;E_{n}-\frac{\xi }{\left(\gamma +\mu \right)}\;B_{C}\right](\;Z+\mu \;E_{n}{)}^{-1}v_{0}$, *and* $R_{1,P}^{\beta }=-R_{0}^{\beta }\frac{1}{\left(\delta +\mu \right)}w_{0}^{T}\Psi v_{0}$.

**Proof.** See Appendix B. □

This result has a number of important consequences as formulated below. We first consider a slightly simplified setup. Let there be no variability in transmission rates, i.e., B_*C*_ = *β_C_* E_*n*_ and ${\bar{B}}_{C}=E_{n}$. Then, according to Lemma 2, the vectors *w*_0_ and *v*_0_ coincide with those of A. In particular, we have *w*_0_ = **1**_[*n*×1]_ since the matrix A is column stochastic. The respective coefficients turn into $R_{0}^{\beta }=R_{0}^{\alpha }$, $R_{1,T}^{\beta }=-R_{0}^{\beta }\frac{\gamma }{\gamma +\xi \mu }w_{0}^{T}(\;Z+\mu \;E_{n}{)}^{-1}v_{0}$, and $R_{1,P}^{\beta }=-R_{0}^{\beta }\frac{1}{\left(\delta +\mu \right)}w_{0}^{T}\Psi v_{0}$. That is, we can write

$$R_{0}^{\beta }(u_{T},u_{P})=R_{0}^{\beta }\left(1-\frac{\gamma }{\gamma +\xi \mu }w_{0}^{T}(\;Z+\mu \;E_{n}{)}^{-1}v_{0}\cdot u_{T}-\frac{1}{\left(\delta +\mu \right)}w_{0}^{T}\Psi v_{0}\cdot u_{P}\right)+O(\|(u_{T},u_{P}){\|}^{2}).$$

Obviously, both controls are unconditionally locally efficient. We can also observe that the control *u_T_* is locally more efficient than *u_P_* if it holds that

(14) $$\frac{\gamma }{\gamma +\xi \mu }w_{0}^{T}(\;Z+\mu \;E_{n}{)}^{-1}v_{0}>\frac{1}{\left(\delta +\mu \right)}w_{0}^{T}\Psi v_{0}.$$

The inequality Equation (14) implies that the control *u*_*T*_ decreases the basic reproduction number to a larger extent, as the respective sensitivity coefficient $R_{1,T}^{\beta }$ is larger in absolute value than $R_{1,P}^{\beta }$. Obviously, we have that *u*_*P*_ is locally more efficient if the opposite holds true. The inequality Equation (14) can be interpreted as follows. Note that *τ_i_* = 1/(*ζ_i_* + *μ*) and *π* = 1/(*δ* + *μ*) are the average duration of being either on treatment or on prophylaxis and recall that $w_{0}^{T}=[1,\;\ldots ,\;1]$. Then we can write Equation (14) as

$$\sum_{i}\frac{\gamma }{\gamma +\xi \mu }\tau_{i}v_{0i}>\sum_{i}\psi_{i}\pi v_{0i}.$$

Here, the factor *γ*/(*γ* + *ξμ*) is interpreted as the degree of protection given by the treatment. Note that this number decreases with increasing *ξ*, i.e., when the acute stage is much more contagious compared to the chronic stage. If *ξ* = 1, the fraction *γ*/(*γ* + *μ*) merely corresponds to the fraction of people that survive to the chronic stage. Note that this interpretation has to do with the fact that we assume the acute stage is short enough that people will not start treatment while they are in the acute stage of infection. Therefore, the duration and contagiousness of the acute stage of infection are potentially strong determinants of the efficacy of therapy as a population-level control. This assumption is reasonable for diseases like HIV, but may need to be revisited for application to other diseases. Next, we note that the components of the vector *v*_0_ are proportional to the stationary distribution of different strains of the virus in the baseline model (see Section 4 for more details on that). Thus, we can interpret the sensitivity parameters $R_{1,T}^{\beta }$ and $R_{1,P}^{\beta }$ as a sum of products *average duration of the medical intervention* × *protection conferred by the intervention* taken with the weights corresponding to the stationary distribution of the virus strains.

Following the same line, one can attempt to compare the efficiency of two controls in the general case. To start with, we write Equation (13) as

$$R_{0}^{\beta }(u_{T},u_{P})=R_{0}^{\beta }\left(1-w_{0}^{T}\left[E_{n}-\frac{\xi }{(\gamma +\mu )R_{0}^{\beta }}B_{C}\right](\;Z+\mu \;E_{n}{)}^{-1}v_{0}u_{T}-\frac{1}{\left(\delta +\mu \right)}w_{0}^{T}\Psi v_{0}u_{P}\right)+O(\|(u_{T},u_{P}){\|}^{2})$$

As above, we say that *u_T_* is locally more efficient than *u_P_* if

(15) $$\sum_{i}\left[1-\frac{\beta_{Ai}\theta_{A}}{R_{0}^{\beta }}\right]\tau_{i}w_{0i}v_{0i}>\sum_{i}\psi_{i}\pi w_{0i}v_{0i},$$

where *β _A*i*_* is the contagiousness of the *i*th strain during the acute stage and *θ_A_* is the average duration of the acute stage (cf. Remark 1).

Similarly to the previous case, we interpret the expression in front of *τ_i_* as the degree of protection given by the treatment to those infected with the i-type virus. Note that a sufficient condition for this expression to be positive is *β*_*Ai*_*θ*_*A*_ < 1. In contrast to the previous case, the components *w*_0*i*_*v*_0*i*_ do not have that clear interpretation. However, their behavior is pretty close to that of *v*_0*i*_.

Finally, we observe that for sufficiently large controls *u_T_* and *u_P_* we have

$$\underset{\begin{array}{c} u_{T}\to \infty \\ u_{P}\to \infty \end{array}}{\text{lim}}R_{0}^{\beta }(u_{T},u_{P})=\frac{\xi }{\left(\gamma +\mu \right)}\rho \;(\;B_{C}\;[\;E_{n}-\Psi ])=\frac{\xi }{\left(\gamma +\mu \right)}\max_{i}(\beta_{Ci}(1-\psi_{i})),$$

which yields the result that agrees with the result of Lemma 1.

**Lemma 3.** *The controls u*_*T*_ *and u*_*P*_ *are jointly globally efficient if*

$$\xi \max_{i}(\beta_{Ci}(1-\psi_{i}))<\gamma +\mu .$$

## 4. Endemic Equilibrium

In contrast to the unique disease-free equilibrium, there can be one, many (a continuum), and no endemic equilibria at all. Which case realizes in our system depends on the value of the basic reproduction number and on the structure of the matrix A as will be shown below. For the general case, the endemic equilibrium can be computed using a rather involved semi-analytic procedure and offers a little insight into the structure of the respective equilibrium. Therefore, we restrict ourselves to the baseline model. The general model is considered in Section 5 that is devoted to the numerical simulations.

We begin by stating a general result on the endemic equilibrium.

**Theorem 4.** *Let A be an irreducible non-negative column stochastic matrix such that all diagonal elements are non-zero. Then the endemic equilibrium for the system* Equation (1) *exists and is unique if R*_0_ > 1. *Let, furthermore, v*^⊤^ = [*v*_1_, … , *v*_*n*_] *be the right normalized eigenvector of* A *corresponding to the dominant eigenvalue of* A *and satisfying* $\sum_{i=1}^{n}v_{i}=1$. *The components of the endemic equilibrium state are given by*

(16) $$I_{Ai}^{*}=\frac{\mu }{\left(\gamma +\mu \right)}\left(1-\frac{1}{R_{0}}\right)v_{i},\;\;\;\;\;I_{Ci}^{*}=\frac{\gamma \mu }{\left(\gamma +\mu )(u_{T}+\mu \right)}\left(1-\frac{1}{R_{0}}\right)v_{i},\;T^{*}=\frac{\gamma u_{T}}{\left(\gamma +\mu )(u_{T}+\mu \right)}\left(1-\frac{1}{R_{0}}\right),\;S^{*}=\frac{1}{R_{0}}.$$

**Proof.** See Appendix B. □

Note that the only additional property of the matrix A that is involved in this theorem is that A is *irreducible.* For the definition of irreducibility and further details see Appendix A.

The obtained result can be used to compute a number of derived quantities. For instance, we have that the total prevalence at the endemic equilibrium is equal to

$$\Pi =1-S^{*}=\frac{R_{0}-1}{R_{0}}$$

and the ratio of transmissions through acutely infected to the transmission through chronically infected is given by

(17) $$r_{AC}=\frac{\xi \beta_{C}\sum_{i=1}^{n}\;I_{Ai}}{\beta_{C}\sum_{i=1}^{n}\;I_{Ci}}=\frac{\xi \beta_{C}i_{A\Sigma }}{\beta_{C}i_{C\Sigma }}=\frac{\xi \beta_{C}(u_{T}+\mu )}{\beta_{C}\gamma }.$$

Using the statistical estimations of these two parameters one can recover *ξ* and *β*_*C*_.

Before we proceed to the next result we recall that *α_ij_* can be interpreted as the probability of catching a virus of type *i* through the contact with an individual initially infected by the virus of type *j*. So, we can make the following observation.

**Lemma 4.** *At the endemic equilibrium, the probability of encountering a chronically infected in the ith category is equal to the probability of catching the type i virus through the contact with a randomly chosen chronically infected individual:*

$$I_{Ci}^{*}=\sum_{j=1}^{n}\alpha_{ij}I_{Cj}^{*}.$$

**Proof.** Using the expression for $I_{Ci}^{*}$ from Equation (16), we can write

$$\sum_{j=1}^{n}\alpha_{ij}I_{Cj}^{*}=\frac{\gamma \mu }{\left(\gamma +\mu )(u_{T}+\mu \right)}\left(1-\frac{1}{R_{0}}\right)\sum_{j=1}^{n}\alpha_{ij}v_{j}.$$

However, since *v* is the dominant eigenvector of A, it holds that $\sum_{j=1}^{n}\alpha_{ij}v_{j}=v_{i}$, whence the result follows. □

If the matrix A is reducible, the results of Theorem 4 do not apply any longer. However, we can formulate a weaker version of the theorem. First, we note that a reducible matrix can be transformed to the normal form by means of a properly chosen permutation matrix:

(18) $$\tilde{A}={\text{PAP}}^{T}=\left[\begin{array}{cccc} {\tilde{A}}_{1} & * & \ldots & *\\ 0 & {\tilde{A}}_{2} & \ldots & *\\ \vdots & & \ddots & *\\ 0 & 0 & \ldots & {\tilde{A}}_{k} \end{array}\right],$$

where A_*i*_, *i* = 1, … , *k* are irreducible matrices and asterisks denote arbitrary non-negative matrices.

**Theorem 5.** *Let* A *be a reducible non-negative matrix with non-zero diagonal elements such that it can be transformed into the normal form* Equation (18) *by an appropriate simultaneous permutation of rows and columns. Then* A *has at most k unit eigenvalues. Furthermore, let* **v** = {*v*_1_, … , *v_q_*} *be the set of normalized eigenvectors corresponding to the unit eigenvalues, q* ≤ *k. Then the set of endemic equilibria is defined as follows:*

(19) $$I_{Ai}^{*}=\frac{\mu }{\left(\gamma +\mu \right)}\left(1-\frac{1}{R_{0}}\right){\bar{v}}_{i},\;\;\;\;\;I_{Ci}^{*}=\frac{\gamma \mu }{\left(\gamma +\mu )(u_{T}+\mu \right)}\left(1-\frac{1}{R_{0}}\right){\bar{v}}_{i},\;T^{*}=\frac{\gamma u}{\left(\gamma +\mu )(u_{T}+\mu \right)}\left(1-\frac{1}{R_{0}}\right),\;S^{*}=\frac{1}{R_{0}},$$

*where the vector* $\bar{v}$ *belongs to the linear hull of vectors from* $v:\bar{v}\in \text{Span}(v)$.

Theorem 5 implies that the set of endemic equilibria can form a linear subspace of the system’s state space. The case when the matrix A is reducible corresponds to the situation when there are some particular groups of virus strains, say, two groups *G*_1_ and *G*_2_. Reducibility implies that the mutations between these groups are either not possible at all, *G*_1_ ↮ *G*_2_ or possible only in one direction, *G*_1_ ← *G*_2_, but *G*_1_ ↛ *G*_2_ (or vice versa). Such a setup allows for considering directed patterns of viral evolution. However, this question is beyond the scope of this paper and will be addressed in our future work.

### Structure of the Matrix A: Uniform within Host Mutations

An important observation that follows from the preceding analysis is that one cannot unambiguously determine all *n*^2^ parameters *α_ij_* from the observations made at the endemic equilibrium. The reason for this is that the equilibrium values depend on the *n* components of the vector *v* (see Equation (16)) of which only *n* – 1 values are independent. We thus restrict ourselves to considering one particular structure of the matrix A that can be formulated in terms of only *n* parameters. More complex structures are possible and can be treated using the same results. In particular, Theorem A1 in Appendix A offers a convenient tool for computing the respective dominant eigenvector.

Assume that during the chronic infection stage the viral population of the individual, initially infected with the type i virus contains the fraction *π_i_* of the original virus while the remaining strains of the virus are distributed uniformly. This means that the matrix A has the following form:

(20) $$A=\left[\begin{array}{cccc} \pi_{1} & \frac{1-\pi_{2}}{n-1} & \ldots & \frac{1-\pi_{n}}{n-1}\\ \frac{1-\pi_{1}}{n-1} & \pi_{2} & & \frac{1-\pi_{n}}{n-1}\\ \vdots & \vdots & \ddots & \vdots \\ \frac{1-\pi_{1}}{n-1} & \frac{1-\pi_{2}}{n-1} & \ldots & \pi_{n} \end{array}\right].$$

To make a connection to the standard form of the matrix A Equation (6) we note that with such parametrization we have *α_ii_* = *π_i_* and $\alpha_{ji}=\frac{1-\pi_{i}}{n-1}$ for all *j* ≠ *i*.

The matrix Equation (20) is positive hence, the Perron–Frobenius theorem applies. There is a unique dominant eigenvalue that is equal to 1, and the components of the dominant eigenvector have the following form:

$$v_{i}=\frac{\prod_{j\neq i}(1-\pi_{j})}{\sum_{i=1}^{n}\prod_{j\neq i}(1-\pi_{j})}.$$

The respective expressions for the system states at endemic equilibrium are pretty bulky. However, we can compute the ratios of infected in different groups, which turn out to have a simple form:

$$r_{ij}=\frac{I_{Ai}+I_{Ci}}{I_{Aj}+I_{Cj}}=\frac{v_{i}}{v_{j}}=\frac{1-\pi_{j}}{1-\pi_{i}}.$$

Note that the condition Σ_*i*_ *v_i_* = 1 implies that there are only (*n* – 1) independent equations. Thus, one parameter *π_i_* can be set to an arbitrary value within the range [0, 1]. Let, for instance, *π_n_* be used as a free parameter. In this case, all remaining probabilities can be expressed in terms of *π_n_* and *v_i_*:

$$\pi_{j}=1-(1-\pi_{n})\frac{v_{n}}{v_{j}},\;\;j=1,\;\ldots ,\;n-1.$$

In the following we will consider a slightly more realistic scenario in which all viruses are ordered according to their genetic similarity and any virus can mutate only to its “neighbors”. The respective matrix A has the following form:

(21) $$A=\left[\begin{array}{cccc} \pi_{1} & \frac{1-\pi_{2}}{2} & \ldots & 0\\ 1-\pi_{1} & \pi_{2} & & 0\\ 0 & \frac{1-\pi_{2}}{2} & \ldots & 0\\ \vdots & \vdots & \ddots & \vdots \\ 0 & 0 & \ldots & \pi_{n} \end{array}\right].$$

Matrix A in Equation (21) is non-negative, irreducible and acyclic. Hence, the Perron–Frobenius theorem applies as well. Quite remarkably, the respective expressions do not change that much compared to the previous case. Setting *v_i_* and assuming that *π_n_* can be freely chosen we get

(22) $$\pi_{1}=1-(1-\pi_{n})\frac{v_{n}}{v_{1}},\;\pi_{j}=1-2(1-\pi_{n})\frac{v_{n}}{v_{j}},\;\;j=2,\;\ldots ,\;n-1.$$

## 5. Numerical Simulation for Different Scenarios and Illustration of Results

To illustrate our results we will present several scenarios that are aimed at illustrating different aspects of the considered problem. As a testbed for our numerical analysis we consider a model with four virus strains. On the one hand, this model is complex enough to illustrate different interesting features of the studied model, but on the other hand, it can be easily visualized and analyzed. We believe that such setup might be a reasonable use case of the framework where we have a limited number of putative strains that are characterized by well-defined phenotypes. A particularly important fact is that our results are valid for any number of strains.

First consider the baseline model (no prophylaxis, single treatment, and uniform contagiousness). Later on we will extend this model along several directions. We take the following values of the parameters: *μ* = 0.025, *γ* = 3 (i.e., the acute phase takes about four months); *ξ* = 5 (during the acute phase an individual is five times more contagious as in the chronic one); *u_T_* = 0.4 (it takes 2.5 years on average until the treatment begins). The baseline transmissibility rate *β_C_* was chosen such that *R*_0_(*u_T_* = 0.4, *u_P_* = 0) ≈ 1.2: *β_C_* = 0.25. The matrix A is assumed to have the form Equation (21). When choosing the values of the probabilities *π_i_*, we imposed the following conditions on the endemic distribution of the different virus strains:

**Case 1.** *v_j_/v*_*j*+1_ = 3, *j* = 1, 2, 3. Assuming that *π*_4_ = 0.25 one can compute the remaining probabilities using Equation (22): *π*_1_ ≈ 0.97, *π*_2_ ≈ 0.83, and *π*_3_ = 0.5. Finally, the endemic frequencies are [*v*_1_, *v*_2_, *v*_3_, *v*_4_] = [0.675, 0.225, 0.075, 0.025].

**Case 2.** *v_j_/v*_*j*+1_ = 7, *j* = 1, 2, 3. Similarly to the previous case, we fix *π*_4_ = 0.25 and compute the remaining probabilities *π*_1_ = 0.9985, *π*_2_ = 0.9796, and *π*_3_ = 0.8571. The respective endemic frequencies are [*v*_1_, *v*_2_, *v*_3_, *v*_4_] = [0.8575, 0.1225, 0.0175, 0.0025].

Now we proceed with a detailed qualitative analysis of the two described models.

### 5.1. Controlled Basic Reproduction Number

**Figure 1.**
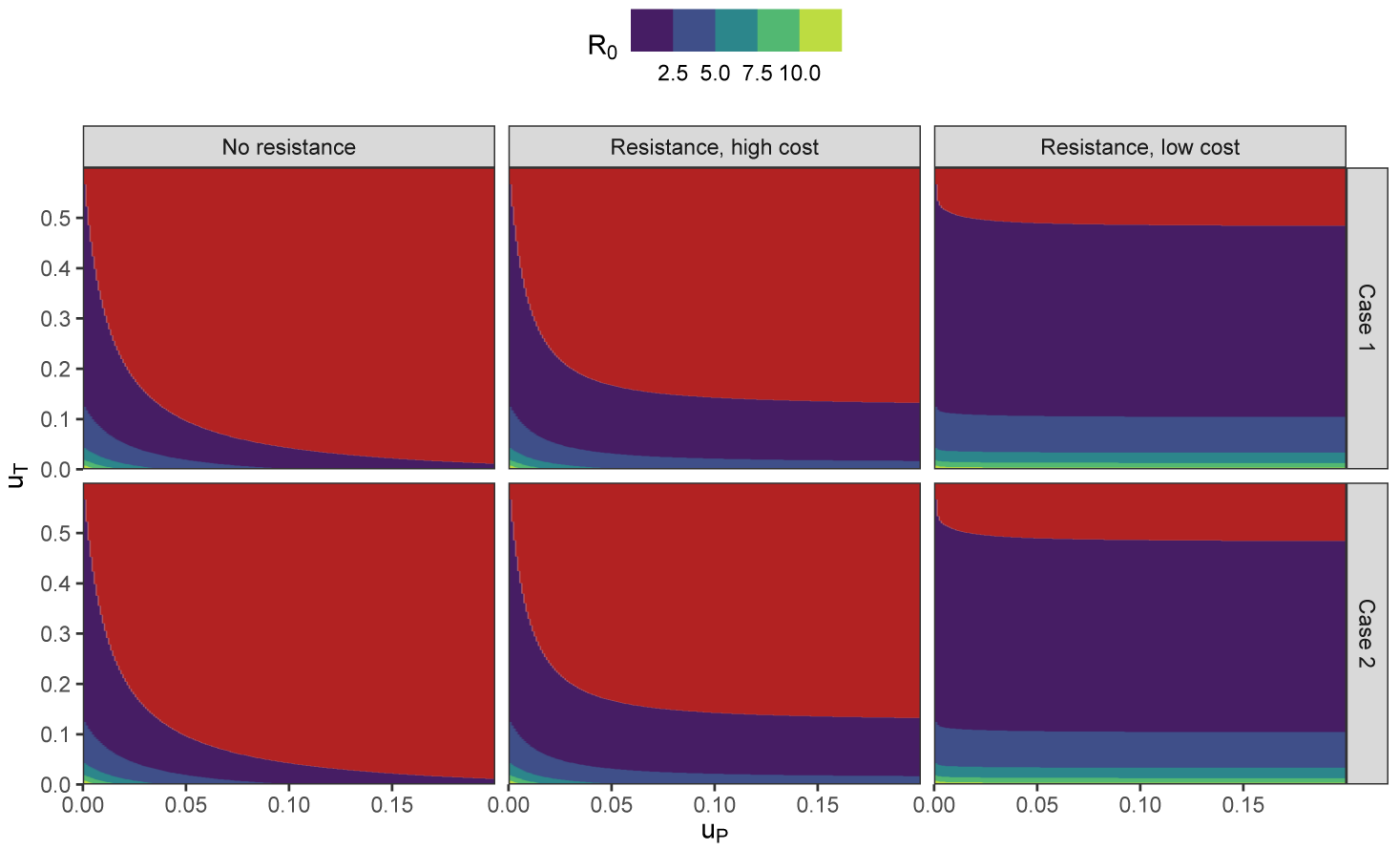
The panel shows the values of *R*_0_ (*u_T_*, *u_P_*) as a function of two controls for two cases described above. The red color corresponds to the case *R*_0_ ≤ 1. We assume a uniform rate of transmission, i.e., *β_i_* = *β* = 0.3 for all *i* = 1, … , 4 and fully efficient treatment, i.e., *ζ_i_* = 0, *i* = 1, … , 4. Remaining parameters are: *ξ* = 5; *γ* = 3; *μ* = 0.025; and *δ* = 0. The subfigures forming the panel correspond to the following values of prophylaxis efficiency coefficients: left, *ψ* = [1, 1, 1, 1]; central, *ψ* = [1, 1, 1, 0]; right, *ψ* = [, 1, 1, 1].

Equation (12) that gives the controlled basic reproduction number for the extended model does not have an immediately apparent intuitive interpretation, however, it allows us to measure the influence of within-host evolution on the controllability of pathogens by therapy and prophylaxis. Figure 1 illustrates how Equation (12) can be used to measure the effects of different levels of resistance to prophylactic interventions on the extent of controls needed to bring a pathogen to sub-critical levels. In the “No resistance” case, we assume that resistance to prophylaxis cannot be evolved (*ψ_i_* = 1, *i* = 1, … , 4), and we see a clear synergistic interaction between the therapy and prophylactic controls; the level of therapeutic control can be reduced by more than half by even a modest investment in the prophylactic control. In the “Resistance, high cost” we assume that *ψ*_4_ = 0, that is, a very rare strain is resistant to the prophylactic control. We refer to this scenario as ‘high cost’ because the strain quickly reverts to a wild-type variant suggesting that the mutations involved have a high evolutionary cost and, in the absence of the prophylactic control, the mutations defining this strain are slightly deleterious. In this scenario, the benefit of increasing the prophylactic control saturates when the selection for the resistant phenotype balances out the deleterious effects of the resistance mutations and there is no further population-level benefit to prophylaxis. Minor variants that confer resistance, even when that resistance is associated with a non-trivial evolutionary cost, can have a major effect on the control properties of the system. In the “Resistance, low cost” scenario we assume that the evolutionary cost of mutations that confer resistance is lower (i.e., once the mutation(s) occur, they tend to be lost at a slower rate), which we parameterize by setting *ψ*_1_ = 0. In this scenario, even a very small level of prophylaxis leads to the resistant strain becoming the dominant strain and has only a small effect on *R*_0_. The results in Case 1 and Case 2 were nearly identical suggesting that the control properties of the system are robust to some level of variation in the underlay within-host evolutionary dynamics. Note that we consider the uniform transmissibility/uniformly efficient treatment case, hence there is a little phenotypic variability in the model.

### 5.2 Endemic Distribution with Variable Transmissibility

To see how varying transmissibility influences the endemic distribution we fix the transmissibilities of the first three strains to be equal to *β_C_*, while the transmissibility of the fourth strain is *β*_*C*4_ = *aβ_C_*, where *a* changes from 0.7 to 2. This particular choice is dictated by the wish to have a well expressed example of the variation of endemic frequencies. Obviously, if we varied the transmissibility of a different strain, the result would be the same, but less expressed.

**Figure 2.**
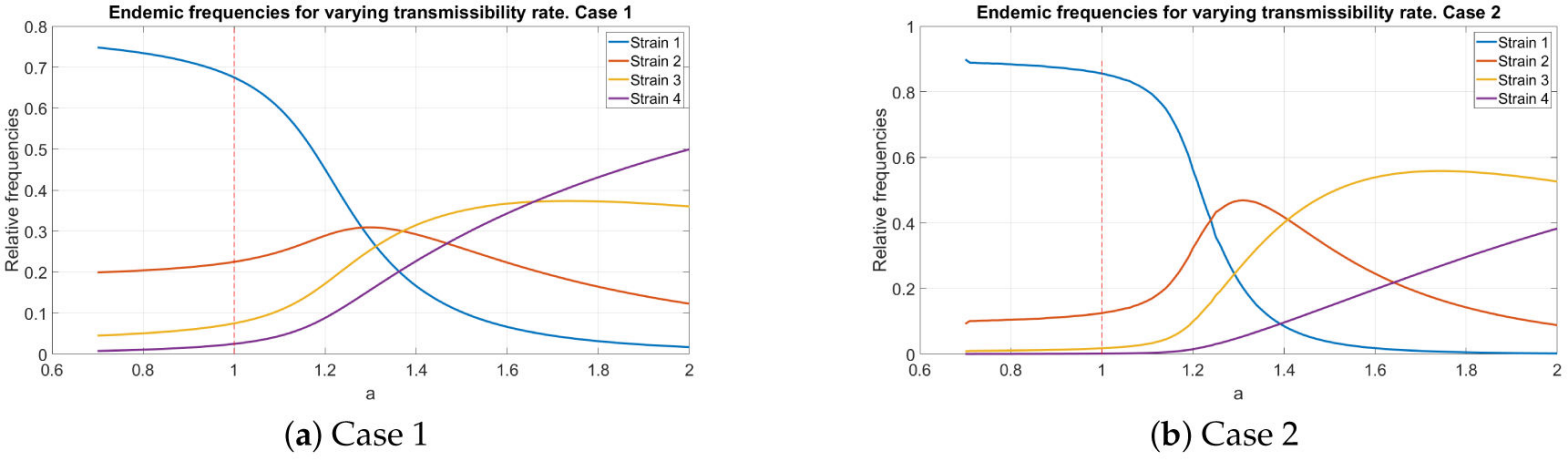
The relative endemic distribution of infected individuals for different values of the transmissibility rate of the 4th strain, parametrized with *a*: *β*_*C*,4_ = *aβ_C_*. The values at *a* = 1 (marked by a red dashed line) correspond to the baseline case, where all transmissibility rates are equal. Subfigures (**a**) and (**b**) correspond to different values of mutation probabilities *π_i_*.

The resulting relative endemic frequencies for both cases are shown in Figure 2. The relative frequencies were computed as $f_{i}=\frac{I_{Ai}+I_{Ci}}{\sum I_{Ai}+\sum I_{Ci}}$. Note that at *a* = 1 the endemic distribution coincides with the baseline one. It is interesting to observe that variation in transmissibility of one strain leads to substantial variation in the frequencies of the other strains as facilitated by within-host mutation.

### 5.3. Endemic Distribution with Variable Prophylaxis Effects

To study the effect of prophylaxis on the endemic distribution of different strains we assume that prophylaxis confers a full protection against the three first strains, while providing no protection against the last strain. That is to say, the matrix Ψ has the form

$$\Psi =\left[\begin{array}{cccc} 1 & 0 & 0 & 0\\ 0 & 1 & 0 & 0\\ 0 & 0 & 1 & 0\\ 0 & 0 & 0 & 0 \end{array}\right].$$

**Figure 3.**
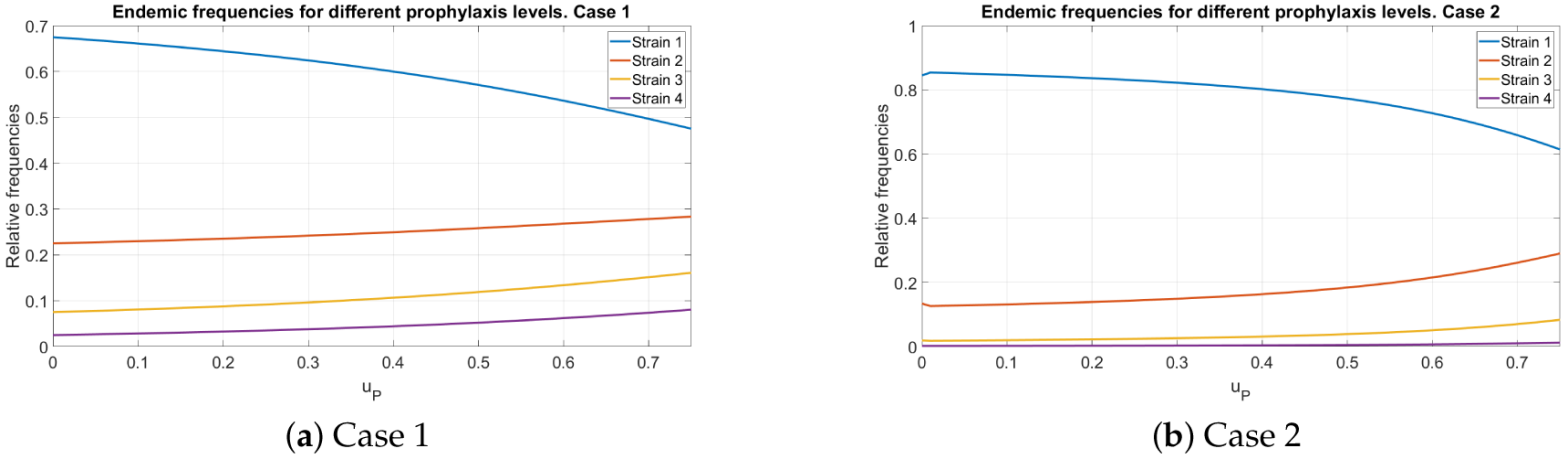
The relative endemic distribution of infected individuals for different values of *u_P_*. Subfigures (**a**) and (**b**) correspond to different values of mutation probabilities *π_i_*.

Furthermore, we assume that the prophylaxis program takes three months, i.e., *δ* = 4. We change the rate at which people are recruited to prophylaxis and study its effect on the relative frequencies of respective strains. The results are presented in Figure 3.

We see that as *u_P_* grows, the fraction of the last, resistant strain grows as well. In general, the frequencies of strains tends to a more uniform distribution. On the other hand, as *u_P_* grows, the total fraction of infected individuals decreases and approaches zero for *u_P_* = 0.88 (Case 1) or *u_P_* = 0.8 (Case 2). It is worth noting that even for a sufficiently large value of *u_P_* = 0.75, the fraction of the total population being on prophylaxis does not exceed 15%. This can be explained by a relatively fast turnover: one cycle of prophylaxis lasts three months, after which the individual returns to the group of susceptible.

This implies that while imperfect prophylaxis leads to some increase in the frequencies of the viruses that evade it, this increase is rather restricted. The main reason is that when prophylaxis covers a small fraction of the population it does not create sufficient evolutionary pressure, while when it increases it eventually contributes to the complete eradication of the disease. This result is potentially very encouraging as new prevention methods for HIV based on administration of broadly neutralizing antibodies are predicted to have highly differential levels of protection to diverse viral panels [17]. Although further work is needed to explore the potential of selective prophylactic agents to cause strain-level selection in populations in the context of within-host mutation.

### 5.4. Endemic Distribution with Imperfect Treatment

**Figure 4.**
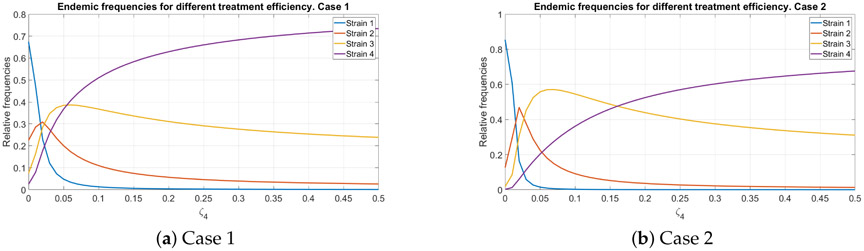
The relative endemic distribution of infected individuals for different values of *ζ*_4_. Subfigures (**a**) and (**b**) correspond to different values of mutation probabilities *π_i_*.

**Figure 5.**
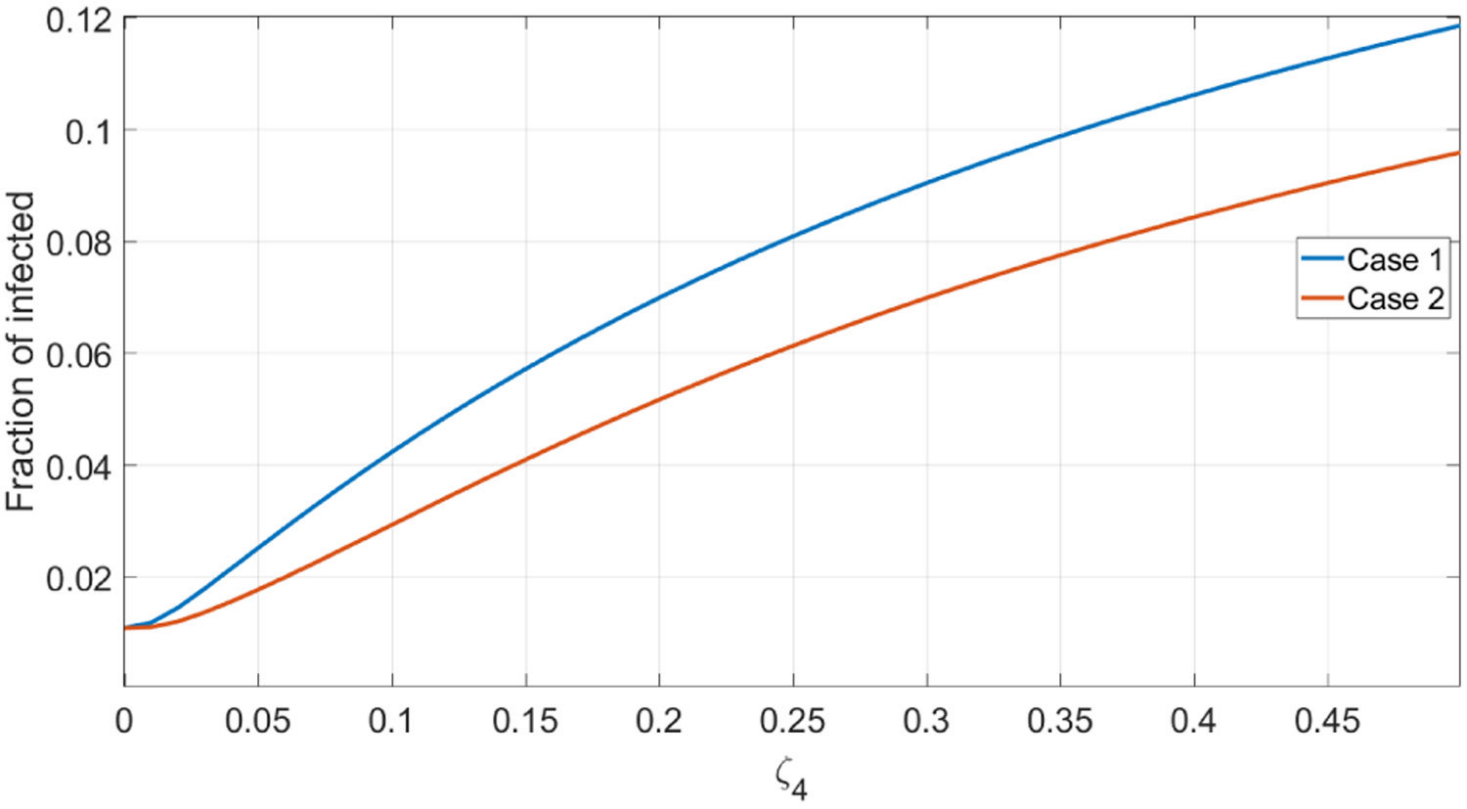
The total fraction of infected as a function of *ζ*_4_.

To study the situation with imperfect treatment we follow the same route and assume that the treatment is absolutely efficient for all strains except the last one. We vary the rate at which the treatment fails and compute the endemic distribution of strains as shown in Figure 4. We also assume that there is no prophylaxis. The result turns out to be quite surprising: not only the endemic frequencies reshuffle, but also the total proportion of infected individuals increases dramatically, see Figure 5.

## 6. Conclusions

In this paper, we described two models of joint evolutionary and epidemiological dynamics of a viral pathogen. While the first baseline model did not take into account the phenotypic variability of the virus, the extended model addressed the within-host evolution among multiple phenotypes characterized by variable contagiousness, resistance to prophylactic measures, and resistance to therapeutic measures. We presented an analytic expression for the controlled basic reproduction number for both cases and carried out sensitivity analysis of the derived expression to the changes of the control actions. It turned out that the sensitivity coefficients $R_{1}^{T}$ and $R_{1}^{P}$ have a straightforward interpretation that can be used when assessing the relative efficacy of the controls. Further, we characterized the endemic equilibria for the baseline model and an extension thereof and showed that a sole assumption of variable transmissibility of different virus strains can lead to wide variations in the endemic distribution of the respective strains. Finally, we carried out a numerical analysis aimed at analyzing the effects of phenotypic diversity of virus strains on the population level dynamics and distribution of different virus strains within the population. Our presented framework can be used as a basic foundation for studying the complex interventions such as imperfect vaccines, antibody-based prophylaxis, and new small-molecule therapeutics for a variety of chronic infections such as HIV, herpes, and HPV. It is possible that this framework may be useful for studying even reality short lived infections such as COVID-19. Early data suggests that the virus accumulates mutations over the course of a single infection [18] and that some of those mutations may affect the viruses’ contagiousness [19]. Likewise, this model could be used to study the implications of strain specific vaccine effects or how different vaccines could change the genetic landscape of SARS-CoV-2.

## Figures and Tables

**Table 1.** Model parameters. Parameters indicated with an asterisk are used only in the extended model Equation (4).

State Variable	Range	Description
*I_Ai_*	[0, 1]	Fraction of acutely infected individuals infected by the virus of type *i*.
*I_Ci_*	[0, 1]	Fraction of chronically infected individuals infected by the virus of type *i*.
*S*	[0, 1]	Fraction of susceptible individuals
*T*	[0, 1]	Fraction of patients involved in treatment
T_i_*	[0, 1]	Fraction of patients infected by the virus of type *i* that are involved in treatment
P*	[0, 1]	Fraction of patients involved in prophylaxis
Parameter	Range	Description
*u_T_*		Rate at which chronically infected are enrolled into treatment (controlled parameter)
u_P_*		Rate at which susceptible individuals are enrolled into prophylaxis (controlled parameter)
*γ*		Inverse duration of the acute phase
*μ*		Mortality rate
*α_ij_*	[0, 1]	Fraction of type *i* viruses in the viral population of an individual initially infected by the type *j* virus.
*β_A_*, *β_C_*		Transmissibility rates of acute and chronically infected individuals.
*ξ*		Proportionality coefficient of the transmissibility in acute and chronic stages
ζ_i_*		Failure rate of treatment for individuals infected by the virus of type *i*
δ*		Failure rate of prophylaxis
ψ_i_*	[0, 1]	The level of protection against the virus strain *i*, which is conferred by prophylaxis; *ψ_i_* = 1 corresponds to full protection

## Appendix

### Appendix A. Necessary Ingredients from Matrix Algebra

In this appendix, we present some facts about non-negative and stochastic matrices that will be used in the sequel. The interested reader can find a thorough treatment of non-negative matrices, in particular the Perron–Frobenius theory in [20]. The theory of stochastic matrices within the context of Markov chains is detailed in [21].

#### Appendix A.1. Non-Negative Matrices

A matrix *M* is said to be *non-negative* (*positive*), denoted by *M* ⪰ 0 (*M* ≻ 0), if it is element-wise non-negative (positive). The matrix *M* is said to be *reducible* if there exists a permutation matrix *P* such that the conjugated matrix *PMP*^⊤^ has a block upper-triangular form. Otherwise the matrix *M* is said to be *irreducible*. The matrix *M* is *primitive* if it is non-negative and there exists k∈N such that *M^k^* ≻ 0. A non-negative irreducible matrix is primitive if at least one diagonal element is non-zero.

For irreducible non-negative matrices, there exists a real eigenvalue, called *dominant* that is equal to the spectral radius of the matrix. The corresponding left and right dominant eigenvectors are positive. This result follows from the celebrated Perron–Frobenius theorem [20]. In a reducible case, the above results should be substantially weakened to remain true. In particular, there can be multiple eigenvalues corresponding to the spectral radius *r* and the respective eigenvectors are merely non-negative, rather than positive. If a non-negative matrix *M* is reducible, it can be transformed to the *normal form*, which corresponds to a block upper diagonal matrix such that the diagonal blocks are irreducible.

#### Appendix A.2. Stochastic Matrices

A non-negative matrix *Q* is said to be *column stochastic* (*row stochastic*) is all its columns (rows) sum to 1. The notions of (ir)reducibility, primitivity and the Perron–Frobenius theorem can be extended to stochastic matrices in a straightforward manner. Below we mention several properties that are specific for stochastic matrices.

A stochastic matrix is typically used to describe the transition structure of a Markov chain. The spectral radius of a stochastic matrix is equal to 1. The respective normalized right eigenvector *v* is called the *stationary distribution* of the respective Markov chain, i.e., *Qv* = *v*. Here, normalization means that the components of *v* must sum to 1. If the stochastic matrix is irreducible, then due to the Perron–Frobenius theorem the stationary distribution is unique and component-wise positive. Finally, we present the result on computing the stationary distribution of an irreducible stochastic matrix. This is a version of the Markov chain tree theorem [22], formulated using the results from matrix theory (cf. [23]).

**Theorem A1.** *Given an* [*n* × *n*] *irreducible column stochastic matrix* Q, *the ith element of the right dominant eigenvector of* Q *is defined as the ith principal minor of the corresponding Laplacian* Λ = Q – E_*n*_*:*

$$w_{i}=[\Lambda {]}_{i,i}.$$

**Proof.** We have Q*w* = *w*, which is equivalent to (Q – E_*n*_)*w* = Λ*w* = 0. That is, *w* is the eigenvector corresponding to the zero eigenvalue of Λ or, alternatively, *w* ∈ ker(Λ). By the Perron–Frobenius theorem, the eigenspace associated with the dominant eigenvalue of *Q*, and hence, the kernel of Λ is one-dimensional.

By the definition of the adjugate, we have adj(Λ)Λ = Λ adj(Λ) = det(Λ)E_*n*_ = **0**_*n*_. This means, in particular, that the columns of adj(Λ) are linearly dependent and proportional to the stationary distribution *v*.

By transposing the first expression we get Λ^⊤^ adj(Λ)^⊤^ = 0. The columns of Λ and hence, the rows of Λ^⊤^ sum to 0, which implies that the kernel of Λ^⊤^ is a one-dimensional space spanned by **1**_[*n*×1]_. This means that each column of adj(Λ)^⊤^ has the form col_*i*_ (adj(Λ)^⊤^) = (−1)^*i*+*i*^[Λ]_*i,i*_ · **1**_[*n*×1]_ = [Λ]_*i,i*_ · **1**_[*n*×1]_. Respectively, each column of adj(Λ) has the form col_*i*_ (adj(Λ)) = ([Λ]_1,1_, … , [Λ]_*n,n*_). This concludes the proof. □

### Appendix B. Proofs

**Proof of Theorem 1.** The Jacobian matrix J(X) of Equation (1) evaluated at the DFE has the form

(A1) $$J(\;X){|}_{{}_{X=X_{DFE}}}=\left[\begin{array}{cccc} (\xi \beta_{C}-(\gamma +\mu ))\;E_{n} & \beta_{C}A & 0_{\left[n\times 1\right]} & 0_{\left[n\times 1\right]}\\ \gamma \;E_{n} & -(u+\mu )\;E_{n} & 0_{\left[n\times 1\right]} & 0_{\left[n\times 1\right]}\\ 0_{\left[1\times n\right]} & u1_{\left[1\times n\right]} & -\mu & 0\\ -\xi \beta_{C}1_{\left[1\times n\right]} & -\beta_{C}1_{\left[1\times n\right]} & 0 & -\mu \end{array}\right]$$

We observe that the stability of Equation (A1) is determined by the eigenvalues of its [2*n* × 2*n*] leading submatrix. As a side remark, we mention that this implies that in our case the computation of *R*_0_ requires considering both I_*A*_ and I_*C*_ as infected states (cf. the discussion at the end of Section 2 in [16]). Following the standard procedure, we split the respective submatrix in two thus obtaining

$$\left[\begin{array}{cc} \xi \beta_{C}\;E_{n} & \beta_{C}A\\ 0_{\left[n\times n\right]} & 0_{\left[n\times n\right]} \end{array}\right]+\left[\begin{array}{cc} -(\gamma +\mu )\;E_{n} & 0_{\left[n\times n\right]}\\ \gamma \;E_{n} & -(u+\mu )\;E_{n} \end{array}\right]=F-V.$$

The basic reproductive number is defined as the spectral radius of the product FV^−1^, i.e., *R*_0_ = *ρ*(FV^−1^). Using the standard result on the block matrix inversion we get

(A2) $$V^{-1}=\frac{1}{\left(\gamma +\mu )(u_{T}+\mu \right)}\left[\begin{array}{cc} (u_{T}+\mu )\;E_{n} & 0\\ \gamma \;E_{n} & (\gamma +\mu )\;E_{n} \end{array}\right],$$

The product FV^−1^ is equal to

$$\frac{\beta_{C}}{\left(\gamma +\mu )(u_{T}+\mu \right)}\left[\begin{array}{cc} \xi (u_{T}+\mu )\;E+\gamma \;A & \gamma \;A\\ 0_{n} & 0_{n} \end{array}\right]$$

and hence, *R*_0_ is found as the spectral radius of the [*n* × *n*] matrix (*γ* + *μ*)^−1^ (*u_T_* + *μ*)^−1^ *β_C_* [*ξ*(*u_T_* + *μ*) E + *γ* A]. We use the well known facts that if *λ* is an eigenvalue of the matrix M, then *aλ* is an eigenvalue of the matrix *a*M and *k* + *λ* is an eigenvalue of the matrix [*k*E + M] for any α ∈ **R**, *k* ∈ **R**. This implies that $R_{0}=\frac{\beta_{C}\gamma }{\left(\gamma +\mu )(u_{T}+\mu \right)}\left(\frac{\xi (u_{T}+\mu )}{\gamma }+\rho (\;A)\right)$. Finally, since A is a column stochastic matrix, it holds that *ρ*(A) = 1 and hence, we obtain Equation (8). □

**Proof of Theorem 2.** The Jacobian matrix of Equation (4) evaluated at the DFE has the form

(A3) $$J(\;X){|}_{{}_{X=X_{DFE}}}=\left[\begin{array}{ccccc} \xi \bar{\Psi }\;B_{C}-(\gamma +\mu )\;E_{n} & \bar{\Psi }\;B_{C}\;A & 0_{n} & 0_{\left[n\times 1\right]} & 0_{\left[n\times 1\right]}\\ \gamma \;E_{n} & -(u_{T}+\mu )\;E_{n} & Z & 0_{\left[n\times 1\right]} & 0_{\left[n\times 1\right]}\\ 0_{n} & u_{T}\;E_{n} & -\mu \;E_{n}-Z & 0_{\left[n\times 1\right]} & 0_{\left[n\times 1\right]}\\ -\xi 1_{\left[1\times n\right]}\;B_{C}S_{DFE} & -1_{\left[1\times n\right]}\;B_{C}\;AS_{DFE} & 0_{\left[1\times n\right]} & -(\mu +u_{P}) & \delta \\ -\xi 1_{\left[1\times n\right]}(\;E_{n}-\Psi )\;B_{C}S_{DFE} & -1_{\left[1\times n\right]}(\;E_{n}-\Psi )\;B_{C}\;AS_{DFE} & 0_{\left[1\times n\right]} & u_{P} & -(\delta +\mu ) \end{array}\right],$$

where $\bar{\Psi }=E_{n}-P_{DFE}\Psi$.

The Jacobian Equation (A3) is a block lower-triangular matrix, of which the bottom-right [2 × 2] block is a negated M-matrix and hence Hurwitz. Thus stability of the DFE is determined by the eigenvalues of the leading [3*n* × 3*n*] submatrix. As a side remark, we mention that this implies that the *T_i_* compartments must be considered as infected. That differs from what we observed in the baseline case and emphasizes the importance of the right choice of the infected compartments (see the discussion at the end of Section 2 in [16]).

Following the standard procedure, we split the respective submatrix in two:

$$\left[\begin{array}{ccc} \xi \bar{\Psi }\;B_{C} & \bar{\Psi }\;B_{C}\;A & 0_{n}\\ 0_{n} & 0_{n} & 0_{n}\\ 0_{n} & 0_{n} & 0_{n} \end{array}\right]+\left[\begin{array}{ccc} -(\gamma +\mu )\;E_{n} & 0_{n} & 0_{n}\\ \gamma \;E_{n} & -(u_{T}+\mu )\;E_{n} & Z\\ 0_{n} & u_{T}\;E_{n} & -\mu \;E_{n}-Z \end{array}\right]=F-V$$

A complete expression for the inverse of V is rather bulky. However, we note that since *R*_0_ is computed as the spectral radius of FV^−1^, we need to compute only those blocks of the inverse that enter the leading [*n* × *n*] submatrix of the product FV^−1^. So, we write

$$V^{-1}=\frac{1}{\gamma +\mu }\left[\begin{array}{ccc} E_{n} & 0_{n} & 0_{n}\\ \frac{\gamma }{\mu }\;\Delta (u_{T}) & * & *\\ {}* & * & * \end{array}\right],$$

where we used asterisks to denote the blocks that are not relevant to our problem. The diagonal matrix Δ(*u_T_*) is defined as

$$\Delta (u_{T})=(\;Z+(\mu +u_{T})\;E_{n}{)}^{-1}(\;Z+\mu \;E_{n}).$$

Finally we compute *R*_0_ to be

$$R_{0}(u_{T},u_{P})=\frac{1}{(\gamma +\mu )\mu }\rho \;(\xi \mu \bar{\Psi }\;B_{C}+\gamma \bar{\Psi }\;B_{C}\;A\Delta (u_{T}))=\frac{{\bar{\beta }}_{C}(\gamma +\xi \mu )}{(\gamma +\mu )\mu }\rho \left({\bar{B}}_{C}\bar{\Psi }\frac{1}{\gamma +\xi \mu }\;(\xi \mu \;E_{n}+\gamma \;A\Delta (u_{T}))\right),$$

which is Equation (12). Note that ${\bar{B}}_{C}$ and $\bar{\Psi }$ are diagonal matrices and therefore commute. □

**Proof of Theorem 3.** The proof is similar to the proof of Theorem 3.4 in [16] and hence will only be sketched (note that there is a typo in Equation (10) in [16]; the correct expression is written next to Equation (12) in [16]). First, we note that $R_{0}^{\beta }=R_{0}^{\beta }(0,0)=\frac{{\bar{\beta }}_{C}(\gamma +\xi \mu )}{(\gamma +\mu )\mu }\rho \;(\;{\bar{B}}_{C}\;\bar{A})$. Computation of $R_{1,T}^{\beta }$ and $R_{1,P}^{\beta }$ coefficients boils down to computing partial derivatives of $R_{0}^{\beta }(u_{T},u_{P})$ with respect to either *u_T_* or *u_P_* at *u_T_* = *u_P_* = 0. Using the same approach as in [16], we get

$$R_{1,T}^{\beta }=\frac{{\bar{\beta }}_{C}(\gamma +\xi \mu )}{(\gamma +\mu )\mu }w_{0}^{T}\;Q(0)N^{\prime }(0)v_{0},$$

$$R_{1,P}^{\beta }=\frac{{\bar{\beta }}_{C}(\gamma +\xi \mu )}{(\gamma +\mu )\mu }w_{0}^{T}\;Q^{\prime }(0)N(0)v_{0}.$$

Noting that $Q(0)=\;{\bar{B}}_{C}$, $N(0)=\;\bar{A}$, Q′(0)=∣dduPQ(uP)∣uP=0=−1δ+μB¯CΨ, and N′(0)=∣dduTN(uT)∣uT=0=−γγ+ξμA(Z+μEn)−1 we obtain

(A4a) $$R_{1,T}^{\beta }=-\frac{{\bar{\beta }}_{C}\gamma }{(\gamma +\mu )\mu }w_{0}^{T}\;{\bar{B}}_{C}\;A(\;Z+\mu \;E_{n}{)}^{-1}v_{0},$$

(A4b) $$R_{1,P}^{\beta }=-\frac{{\bar{\beta }}_{C}(\gamma +\xi \mu )}{(\delta +\mu )(\gamma +\mu )\mu }w_{0}^{T}\;{\bar{B}}_{C}\;\Psi \;\bar{A}v_{0}.$$

The expressions Equations (A4a) and (A4b) can be further transformed using the fact that *w*_0_ and *v*_0_ are the left and the right eigenvectors of ${\bar{B}}_{C}\;\bar{A}$ corresponding to the spectral radius of this matrix and expressing $\rho \;(\;{\bar{B}}_{C}\;\bar{A})$ through the basic reproduction number $R_{0}^{\beta }$ as shown below.

$$R_{1,T}^{\beta }=-\frac{{\bar{\beta }}_{C}}{(\gamma +\mu )\mu }w_{0}^{T}\;{\bar{B}}_{C}[(\gamma +\xi \mu )\;\bar{A}-\xi \mu \;E_{n}](\;Z+\mu \;E_{n}{)}^{-1}v_{0}\;\;=-\frac{{\bar{\beta }}_{C}(\gamma +\xi \mu )}{(\gamma +\mu )\mu }w_{0}^{T}\rho (\;{\bar{B}}_{C}\;\bar{A})(\;Z+\mu \;E_{n}{)}^{-1}v_{0}+\frac{{\bar{\beta }}_{C}\xi }{\left(\gamma +\mu \right)}w_{0}^{T}\;{\bar{B}}_{C}(\;Z+\mu \;E_{n}{)}^{-1}v_{0}\;\;=-R_{0}^{\beta }w_{0}^{T}\;(\;Z+\mu \;E_{n}{)}^{-1}v_{0}+\frac{{\bar{\beta }}_{C}\xi }{\left(\gamma +\mu \right)}w_{0}^{T}\;{\bar{B}}_{C}(\;Z+\mu \;E_{n}{)}^{-1}v_{0}\;\;=-w_{0}^{T}\left[R_{0}^{\beta }\;E_{n}-\frac{\xi }{\left(\gamma +\mu \right)}\;B_{C}\right](\;Z+\mu \;E_{n}{)}^{-1}v_{0}$$

$$R_{1,P}^{\beta }=-\frac{{\bar{\beta }}_{C}(\gamma +\xi \mu )}{(\delta +\mu )(\gamma +\mu )\mu }w_{0}^{T}\;\Psi \;{\bar{B}}_{C}\;\bar{A}v_{0}=-\frac{{\bar{\beta }}_{C}(\gamma +\xi \mu )}{(\delta +\mu )(\gamma +\mu )\mu }w_{0}^{T}\;\Psi \rho \;({\bar{B}}_{C}\;\bar{A})\;v_{0}=-R_{0}^{\beta }\frac{1}{\left(\delta +\mu \right)}w_{0}^{T}\Psi v_{0}$$

**Proof of Theorem 4.** When computing the endemic equilibrium we let the equilibrium value of *S* be equal to some (not yet known) value *S**. The respective equilibrium values of *I_Ai_* and *I_Ci_* are found as the solution of the following system of 2*n* algebraic equations:

(A5) $$0=\xi \beta_{C}S^{*}\;I_{A}+\beta_{C}S^{*}\;A\;I_{C}-(\gamma +\mu )\;I_{A}\;0=\gamma \;I_{A}-(u+\mu )\;I_{C},$$

From the first equation we get

$$I_{A}^{*}=\frac{\beta_{C}S^{*}}{(\gamma +\mu )-\xi \beta_{C}S^{*}}\;A\;I_{C}^{*}.$$

Expressing $I_{C}^{*}$ from the second equation of Equation (A5) and substituting into the above equation we obtain the expression for $I_{A}^{*}$:

(A6) $$\left[\frac{\left((\gamma +\mu )-\xi \beta_{C}S^{*})(u_{T}+\mu \right)}{\beta_{C}\gamma S^{*}}\;E_{n}-A\right]I_{A}^{*}=\Gamma \;I_{A}^{*}=0,$$

that is $I_{A}^{*}$ belongs to the kernel of the matrix $\Gamma -\frac{\left((\gamma +\mu )-\xi \beta_{C}S^{*})(u_{T}+\mu \right)}{\beta_{C}\gamma S^{*}}\;E_{n}-A$. The matrix A is non-singular, thus Γ has a non-trivial kernel only if the factor in front of E_*n*_ is equal to one of the eigenvalues of A. The solution $I_{A}^{*}$ would be then equal (up to a positive factor) to the corresponding eigenvector. However, as follows from the Perron–Frobenius theorem, the only positive eigenvector corresponds to its dominant eigenvalue, which is equal to 1 for a stochastic matrix. Any other eigenvector would contain negative components, which contradict the assumption that all system states are non-negative. This implies that the equilibrium value of *S* must satisfy the equation $\frac{\left((\gamma +\mu )-\xi \beta_{C}S^{*})(u_{T}+\mu \right)}{\beta_{C}\gamma S^{*}}-1=0$, whence we obtain

(A7) $$S^{*}=\frac{\left(\gamma +\mu )(u_{T}+\mu \right)}{\xi \beta_{C}(u_{T}+\mu )+\beta_{C}\gamma }=\frac{1}{R_{0}}.$$

The equilibrium solution $I_{A}^{*}$ corresponds to the right dominant eigenvector of the column stochastic matrix A. Since this eigenvector is defined up to the multiplication by a positive scalar, we can further specify it using the following argument. Let *i*_*A*Σ_ be the sum of all components of I_*A*_, i.e., $i_{A\Sigma }=\sum_{j=1}^{n}I_{Ai}^{*}$. Similarly, we write $i_{C\Sigma }=\frac{\gamma }{\left(u_{T}+\mu \right)}i_{A\Sigma }$ and subsequently, we get $T^{*}=\frac{\gamma u}{(u_{T}+\mu )\mu }i_{A\Sigma }$. Since all states sum to 1, we have the following equation

$$i_{A\Sigma }+\frac{\gamma }{\left(u_{T}+\mu \right)}i_{A\Sigma }+\frac{\gamma u}{(u_{T}+\mu )\mu }i_{A\Sigma }+\frac{1}{R_{0}}=1,$$

whence we get

(A8) $$i_{A\Sigma }=\frac{\mu }{\left(\gamma +\mu \right)}\left(1-\frac{1}{R_{0}}\right),i_{C\Sigma }-\frac{\gamma \mu }{\left(\gamma +\mu )(u_{T}+\mu \right)}\left(1-\frac{1}{R_{0}}\right),T^{*}=\frac{\gamma u}{\left(\gamma +\mu )(u_{T}+\mu \right)}\left(1-\frac{1}{R_{0}}\right).$$

Let *v* be the (normalized) dominant eigenvector of A such that $\sum_{i=1}^{n}v_{i}=1$. Multiplying the components of *v* with the respective factors we obtain the expressions for *I_Ai_* and *I_Ci_*.

Finally, we observe that *R*_0_ < 1 implies that the respective components of the endemic equilibrium state turn negative, which implies that there is no admissible endemic equilibrium. This concludes the proof. □
